# [μ-1,2-Bis(4-pyrid­yl)ethene-κ^2^ *N*:*N*′]bis­[aqua­(pyridine-2,6-dicarboxyl­ato-κ^3^ *O* ^2^,*N*,*O* ^6^)copper(II)] dihydrate

**DOI:** 10.1107/S1600536811018411

**Published:** 2011-05-20

**Authors:** Shie Fu Lush

**Affiliations:** a General Education Center, Yuanpei University, No. 306 Yuanpei St, HsinChu 30015, Taiwan

## Abstract

In the title dinuclear Cu^II^ complex, [Cu_2_(C_7_H_3_NO_4_)_2_(C_12_H_10_N_2_)(H_2_O)_2_]·2H_2_O, the water-coordinated Cu^II^ cation is *O*,*N*,*O*′-chelated by a pyridine-2,6-dicarboxyl­ate (pdc) dianion, and one pyridine N atom from a 1,2-bis­(4-pyrid­yl)ethene ligand coordinates to the Cu^II^ cation, completing the CuN_2_O_3_ distorted square-pyriamidial geometry. The Cu—O_water_ bond [2.388 (4) Å] in the axial direction is much longer than the other Cu—O bonds. The 1,2-bis­(4-pyrid­yl)ethene ligand is located across an inversion center with the mid-point of the C=C bond at the inversion center, and bridges two Cu^II^ cations, generating a centrosymmetric dinuclear complex. The crystal structure is stabilized by classical O—H⋯O and weak C—H⋯O hydrogen bonds.

## Related literature

For related Cu^II^ complexes with pyridine-2,6-dicarboxyl­ate ligands, see: Chaigneau *et al.* (2004 ▶); Dong *et al.* (2010 ▶); Ghosh *et al.* (2004 ▶).
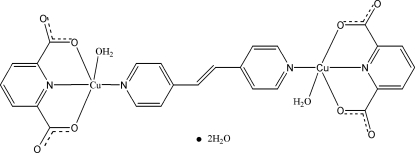


## Experimental

### 

#### Crystal data

[Cu_2_(C_7_H_3_NO_4_)_2_(C_12_H_10_N_2_)(H_2_O)_2_]·2H_2_O
*M* *_r_* = 711.59
Triclinic, 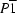

*a* = 5.2616 (5) Å
*b* = 7.9316 (7) Å
*c* = 16.8063 (14) Å
α = 89.183 (2)°
β = 84.541 (2)°
γ = 72.557 (2)°
*V* = 666.01 (10) Å^3^
*Z* = 1
Mo *K*α radiation
μ = 1.67 mm^−1^
*T* = 295 K
0.25 × 0.10 × 0.10 mm

#### Data collection

Bruker SMART 1000 CCD area-detector diffractometer
Absorption correction: multi-scan (*SADABS*; Bruker, 2001 ▶) *T*_min_ = 0.921, *T*_max_ = 0.976
5755 measured reflections
2373 independent reflections
2174 reflections with *I* > 2σ(*I*)
*R*_int_ = 0.036

#### Refinement

*R*[*F* ^2^ > 2σ(*F* ^2^)] = 0.054
*wR*(*F* ^2^) = 0.126
*S* = 1.23
2373 reflections
200 parameters
H-atom parameters constrained
Δρ_max_ = 0.57 e Å^−3^
Δρ_min_ = −0.60 e Å^−3^

### 

Data collection: *SMART* (Bruker, 2007 ▶); cell refinement: *SAINT* (Bruker, 2007 ▶); data reduction: *SAINT*; program(s) used to solve structure: *SHELXTL* (Sheldrick, 2008 ▶); program(s) used to refine structure: *SHELXTL*; molecular graphics: *PLATON* (Spek, 2009 ▶); software used to prepare material for publication: *PLATON*.

## Supplementary Material

Crystal structure: contains datablocks global, I. DOI: 10.1107/S1600536811018411/xu5212sup1.cif

Structure factors: contains datablocks I. DOI: 10.1107/S1600536811018411/xu5212Isup2.hkl

Additional supplementary materials:  crystallographic information; 3D view; checkCIF report

## Figures and Tables

**Table 1:** Selected bond lengths (Å)

Cu1—O1	2.388 (4)
Cu1—O2	2.053 (3)
Cu1—O4	2.003 (4)
Cu1—N1	1.902 (3)
Cu1—N2	1.951 (4)

**Table 2:** Hydrogen-bond geometry (Å, °)

*D*—H⋯*A*	*D*—H	H⋯*A*	*D*⋯*A*	*D*—H⋯*A*
O1—H1*A*⋯O6	0.82	2.10	2.669 (8)	126
O1—H1*B*⋯O2^i^	0.82	1.99	2.809 (5)	175
O6—H6*A*⋯O3^i^	0.82	2.31	2.919 (9)	132
O6—H6*B*⋯O3^ii^	0.82	2.06	2.851 (8)	163
C2—H2*A*⋯O1^iii^	0.93	2.54	3.348 (6)	146
C4—H4*A*⋯O3^iv^	0.93	2.52	3.411 (6)	160
C8—H8*A*⋯O1^v^	0.93	2.49	3.381 (6)	161
C9—H9*A*⋯O5^vi^	0.93	2.47	3.382 (6)	167
C13—H13*A*⋯O5^vii^	0.93	2.35	3.265 (6)	166

Symmetry codes: (i) 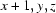; (ii) 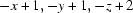; (iii) 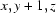; (iv) 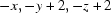; (v) 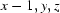; (vi) 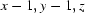; (vii) 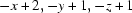.

**Fig. 1.**
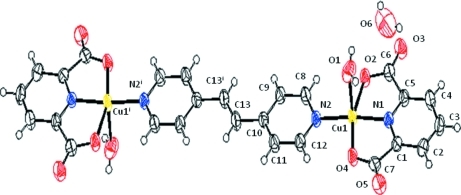
View of the title compound with the atom numbering scheme. Displacement ellipsoids for non-H atoms are drawn at the 50% probability level.[symmetry code: (i) 1 - x, -y, 1 - z].

**Fig. 2.**
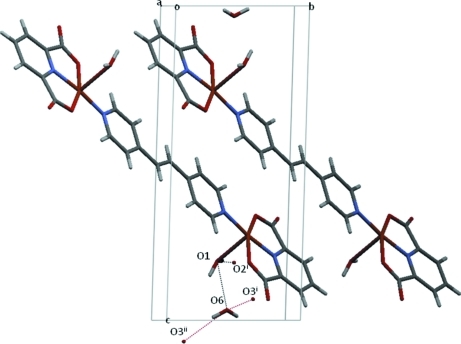
The molecular packing for the title compound. Hydrogen bonds are shown as dashed lines.

## supplementary crystallographic information

### Comment

The pyridine-2,6-dicarboxylic acid (pdcH_2_) has important coordination functions to transition metals by either carboxylate bridges between metal centers, to form dimeric complexes or tridentate (O, N, O') chelation to one metal ion. Some Cu^II^ pdc complexes have been reported (Chaigneau *et al.*, 2004; Ghosh *et al.*, 2004; Dong *et al.*, 2010).

In the title compound, [Cu_2_(C_12_H_10_N_2_)(C_7_H_3_NO_4_)_2_(H_2_O)_2_].2(H_2_O)], the Cu^II^ atom is coordinated by two oxygen atoms and one nitrogen atom of one pyridine-2,6-dicarboxylate (pdc) ligand, one pyrinyl N atom of the 1,2-bis(4-pyridyl)ethene ligand. The distorted square-pyriamidial geometry is completed by a longer axial bond to the O atom of a water molecule [Cu—O 2.390 (43) Å in the axial direction]. The Cu1—N2—N2^i^—Cu1^i^ torsion angle is 180.0 (13)°, assemblies exhibiting *M*—anti-1,2-bis(4-pyridyl)ethene—*M* bridges. Two Cu^II^ atoms are bridged by one *trans*-1,2-bis(4-pyridyl)ethene ligand, generating a dinuclear molecule.The dinuclear molecule is located on a centre of inversion, which is in the middle of the ethylyne fragment of the bpe ligand.

The molecular structure and packing are stabilized by strong O—H···O and weak C—H···O hydrogen bonds, also including a crystal water molecule.

### Experimental

A solution of Cu(NO_3_)_2_.6H_2_O (0.296 g, 1 mmol) in 5 ml H_2_O was added to pyridine-2,6-dicarboxylic acid (0.167, 1 mmol) and 1,2-bis(4-pyridyl)ethane (0.184 g, 1 mmol) in a Teflon-lined stainless steel autoclave which was heated under autogenous pressure to 453 K for 72 h and then allowed to cool to room temperature. Blue columnar crystals of the title compound were collected in 42.35% yield (based on Cu).

### Refinement

Water H atoms were placed in calculated positions and refined with the distance constrains of O—H = 0.82, and *U*_iso_(H)= 1.5*U*_eq_(O). Other H atoms were positioned geometrically with C—H = 0.93 Å, and refined using a riding model with U_iso_(H) = 1.2U_eq_(C).

### Crystal data

**Table:**

[Cu_2_(C_7_H_3_NO_4_)_2_(C_12_H_10_N_2_)(H_2_O)_2_]·2H_2_O	*Z* = 1
*M**_r_* = 711.59	*F*(000) = 362
Triclinic, *P*1	*D*_x_ = 1.774 Mg m^−^^3^
Hall symbol: -P 1	Mo *K*α radiation, λ = 0.71073 Å
*a* = 5.2616 (5) Å	Cell parameters from 3226 reflections
*b* = 7.9316 (7) Å	θ = 2.5–25.0°
*c* = 16.8063 (14) Å	µ = 1.67 mm^−^^1^
α = 89.183 (2)°	*T* = 295 K
β = 84.541 (2)°	Columnar, blue
γ = 72.557 (2)°	0.25 × 0.10 × 0.10 mm
*V* = 666.01 (10) Å^3^	

### Data collection

**Table:**

Bruker SMART 1000 CCD area-detector diffractometer	2373 independent reflections
Radiation source: fine-focus sealed tube	2174 reflections with *I* > 2σ(*I*)
graphite	*R*_int_ = 0.036
Detector resolution: 9 pixels mm^-1^	θ_max_ = 25.1°, θ_min_ = 1.2°
φ and ω scans	*h* = −6→6
Absorption correction: multi-scan (*SADABS*; Bruker, 2001)	*k* = −8→9
*T*_min_ = 0.921, *T*_max_ = 0.976	*l* = −19→19
5755 measured reflections	

### Refinement

**Table:**

Refinement on *F*^2^	Primary atom site location: structure-invariant direct methods
Least-squares matrix: full	Secondary atom site location: difference Fourier map
*R*[*F*^2^ > 2σ(*F*^2^)] = 0.054	Hydrogen site location: inferred from neighbouring sites
*wR*(*F*^2^) = 0.126	H-atom parameters constrained
*S* = 1.23	*w* = 1/[σ^2^(*F*_o_^2^) + (0.0544*P*)^2^ + 0.7371*P*] where *P* = (*F*_o_^2^ + 2*F*_c_^2^)/3
2373 reflections	(Δ/σ)_max_ = 0.002
200 parameters	Δρ_max_ = 0.56 e Å^−^^3^
0 restraints	Δρ_min_ = −0.60 e Å^−^^3^

### Special details

**Table:**

Geometry. Bond distances, angles *etc*. have been calculated using the rounded fractional coordinates. All su's are estimated from the variances of the (full) variance-covariance matrix. The cell e.s.d.'s are taken into account in the estimation of distances, angles and torsion angles
Refinement. Refinement on *F*^2^ for ALL reflections except those flagged by the user for potential systematic errors. Weighted *R*-factors *wR* and all goodnesses of fit *S* are based on *F*^2^, conventional *R*-factors *R* are based on *F*, with *F* set to zero for negative *F*^2^. The observed criterion of *F*^2^ > σ(*F*^2^) is used only for calculating -*R*-factor-obs *etc*. and is not relevant to the choice of reflections for refinement. *R*-factors based on *F*^2^ are statistically about twice as large as those based on *F*, and *R*-factors based on ALL data will be even larger.

### Fractional atomic coordinates and isotropic or equivalent isotropic displacement parameters (Å^2^)

**Table:**

	*x*	*y*	*z*	*U*_iso_*/*U*_eq_	
Cu1	0.57285 (11)	0.65304 (7)	0.73016 (3)	0.0351 (2)	
O1	0.8836 (7)	0.4653 (5)	0.8122 (2)	0.0583 (14)	
O2	0.2784 (6)	0.6365 (4)	0.81661 (17)	0.0396 (10)	
O3	0.0696 (7)	0.7621 (5)	0.9326 (2)	0.0557 (12)	
O4	0.8504 (7)	0.7395 (4)	0.66711 (17)	0.0435 (11)	
O5	1.0829 (8)	0.9308 (5)	0.6771 (2)	0.0616 (16)	
N1	0.5505 (7)	0.8520 (4)	0.7953 (2)	0.0316 (11)	
N2	0.5692 (7)	0.4684 (5)	0.6552 (2)	0.0343 (11)	
C1	0.7125 (9)	0.9487 (6)	0.7732 (2)	0.0355 (14)	
C2	0.7019 (10)	1.0950 (6)	0.8175 (3)	0.0458 (17)	
C3	0.5179 (11)	1.1389 (7)	0.8849 (3)	0.0520 (17)	
C4	0.3530 (10)	1.0343 (7)	0.9072 (3)	0.0478 (17)	
C5	0.3772 (9)	0.8890 (6)	0.8601 (3)	0.0363 (12)	
C6	0.2256 (9)	0.7535 (6)	0.8725 (3)	0.0394 (14)	
C7	0.8995 (10)	0.8702 (6)	0.6991 (3)	0.0406 (16)	
C8	0.4106 (10)	0.3645 (6)	0.6710 (3)	0.0431 (16)	
C9	0.4046 (10)	0.2307 (6)	0.6218 (3)	0.0409 (16)	
C10	0.5697 (9)	0.1958 (6)	0.5501 (2)	0.0354 (14)	
C11	0.7338 (10)	0.3030 (7)	0.5338 (3)	0.0453 (16)	
C12	0.7297 (10)	0.4350 (6)	0.5863 (3)	0.0434 (16)	
C13	0.5754 (10)	0.0543 (6)	0.4941 (3)	0.0486 (17)	
O6	0.7321 (17)	0.5326 (10)	0.9675 (4)	0.149 (4)	
H1A	0.89390	0.41550	0.85540	0.0880*	
H1B	0.99980	0.51510	0.81040	0.0880*	
H2A	0.81460	1.16320	0.80290	0.0550*	
H3A	0.50510	1.23890	0.91520	0.0620*	
H4A	0.23090	1.06180	0.95240	0.0570*	
H8A	0.29840	0.38510	0.71840	0.0520*	
H9A	0.29080	0.16300	0.63600	0.0490*	
H11A	0.84750	0.28520	0.48670	0.0540*	
H12A	0.84250	0.50410	0.57390	0.0520*	
H13A	0.69100	0.03930	0.44750	0.066 (17)*	
H6A	0.78230	0.61090	0.98600	0.2230*	
H6B	0.81680	0.44330	0.98950	0.2230*	

### Atomic displacement parameters (Å^2^)

**Table:**

	*U*^11^	*U*^22^	*U*^33^	*U*^12^	*U*^13^	*U*^23^
Cu1	0.0427 (3)	0.0384 (3)	0.0323 (3)	−0.0274 (2)	0.0089 (2)	−0.0139 (2)
O1	0.052 (2)	0.048 (2)	0.082 (3)	−0.0273 (17)	0.0001 (18)	−0.0071 (18)
O2	0.0426 (18)	0.0427 (17)	0.0397 (16)	−0.0255 (14)	0.0094 (13)	−0.0145 (13)
O3	0.058 (2)	0.063 (2)	0.050 (2)	−0.0321 (18)	0.0239 (17)	−0.0156 (17)
O4	0.056 (2)	0.0483 (19)	0.0359 (16)	−0.0341 (16)	0.0111 (14)	−0.0150 (14)
O5	0.075 (3)	0.075 (3)	0.054 (2)	−0.059 (2)	0.0196 (18)	−0.0117 (18)
N1	0.0359 (19)	0.0311 (18)	0.0327 (18)	−0.0186 (16)	0.0019 (15)	−0.0067 (14)
N2	0.042 (2)	0.0353 (19)	0.0320 (18)	−0.0232 (17)	0.0040 (15)	−0.0094 (15)
C1	0.044 (3)	0.033 (2)	0.036 (2)	−0.022 (2)	−0.0013 (19)	−0.0021 (18)
C2	0.057 (3)	0.039 (3)	0.051 (3)	−0.028 (2)	−0.008 (2)	−0.003 (2)
C3	0.065 (3)	0.040 (3)	0.056 (3)	−0.025 (2)	0.002 (2)	−0.019 (2)
C4	0.054 (3)	0.049 (3)	0.041 (3)	−0.018 (2)	0.004 (2)	−0.018 (2)
C5	0.039 (2)	0.037 (2)	0.037 (2)	−0.0180 (19)	−0.0002 (18)	−0.0092 (18)
C6	0.038 (2)	0.044 (3)	0.041 (2)	−0.022 (2)	0.0053 (19)	−0.008 (2)
C7	0.052 (3)	0.045 (3)	0.034 (2)	−0.031 (2)	0.004 (2)	−0.0013 (19)
C8	0.050 (3)	0.047 (3)	0.037 (2)	−0.026 (2)	0.012 (2)	−0.015 (2)
C9	0.050 (3)	0.041 (3)	0.041 (2)	−0.031 (2)	0.008 (2)	−0.0117 (19)
C10	0.043 (3)	0.036 (2)	0.031 (2)	−0.018 (2)	−0.0008 (18)	−0.0051 (18)
C11	0.053 (3)	0.051 (3)	0.038 (2)	−0.030 (2)	0.015 (2)	−0.015 (2)
C12	0.052 (3)	0.047 (3)	0.040 (2)	−0.031 (2)	0.007 (2)	−0.011 (2)
C13	0.063 (3)	0.049 (3)	0.041 (3)	−0.033 (2)	0.015 (2)	−0.020 (2)
O6	0.195 (7)	0.159 (7)	0.095 (4)	−0.063 (6)	0.008 (5)	−0.015 (4)

### Geometric parameters (Å, °)

**Table:**

Cu1—O1	2.388 (4)	C2—C3	1.394 (7)
Cu1—O2	2.053 (3)	C3—C4	1.394 (8)
Cu1—O4	2.003 (4)	C4—C5	1.375 (7)
Cu1—N1	1.902 (3)	C5—C6	1.520 (7)
Cu1—N2	1.951 (4)	C8—C9	1.364 (7)
O2—C6	1.281 (6)	C9—C10	1.396 (6)
O3—C6	1.229 (6)	C10—C11	1.390 (7)
O4—C7	1.278 (6)	C10—C13	1.467 (6)
O5—C7	1.226 (7)	C11—C12	1.373 (7)
O1—H1A	0.8200	C13—C13^i^	1.336 (7)
O1—H1B	0.8200	C2—H2A	0.9300
O6—H6A	0.8200	C3—H3A	0.9300
O6—H6B	0.8200	C4—H4A	0.9300
N1—C1	1.333 (6)	C8—H8A	0.9300
N1—C5	1.328 (6)	C9—H9A	0.9300
N2—C12	1.346 (6)	C11—H11A	0.9300
N2—C8	1.345 (6)	C12—H12A	0.9300
C1—C2	1.372 (6)	C13—H13A	0.9300
C1—C7	1.526 (6)		
			
O1—Cu1—O2	86.70 (12)	O3—C6—C5	119.9 (4)
O1—Cu1—O4	94.17 (13)	O2—C6—O3	125.8 (4)
O1—Cu1—N1	90.56 (14)	O2—C6—C5	114.3 (4)
O1—Cu1—N2	96.13 (14)	O4—C7—C1	114.4 (4)
O2—Cu1—O4	161.23 (12)	O5—C7—C1	119.4 (4)
O2—Cu1—N1	79.81 (14)	O4—C7—O5	126.1 (5)
O2—Cu1—N2	101.12 (14)	N2—C8—C9	124.0 (5)
O4—Cu1—N1	81.43 (14)	C8—C9—C10	119.8 (5)
O4—Cu1—N2	97.44 (14)	C11—C10—C13	120.6 (4)
N1—Cu1—N2	173.29 (15)	C9—C10—C11	116.3 (4)
Cu1—O2—C6	114.6 (3)	C9—C10—C13	123.1 (4)
Cu1—O4—C7	114.6 (3)	C10—C11—C12	120.7 (5)
H1A—O1—H1B	104.00	N2—C12—C11	122.7 (5)
Cu1—O1—H1B	101.00	C10—C13—C13^i^	124.0 (5)
Cu1—O1—H1A	143.00	C1—C2—H2A	121.00
H6A—O6—H6B	104.00	C3—C2—H2A	121.00
C1—N1—C5	122.9 (4)	C4—C3—H3A	120.00
Cu1—N1—C5	119.5 (3)	C2—C3—H3A	120.00
Cu1—N1—C1	117.7 (3)	C3—C4—H4A	121.00
Cu1—N2—C12	122.0 (3)	C5—C4—H4A	121.00
C8—N2—C12	116.6 (4)	C9—C8—H8A	118.00
Cu1—N2—C8	121.4 (3)	N2—C8—H8A	118.00
N1—C1—C2	120.0 (4)	C8—C9—H9A	120.00
N1—C1—C7	111.5 (4)	C10—C9—H9A	120.00
C2—C1—C7	128.5 (4)	C12—C11—H11A	120.00
C1—C2—C3	118.3 (5)	C10—C11—H11A	120.00
C2—C3—C4	120.5 (5)	N2—C12—H12A	119.00
C3—C4—C5	117.7 (5)	C11—C12—H12A	119.00
N1—C5—C4	120.6 (4)	C10—C13—H13A	118.00
N1—C5—C6	111.7 (4)	C13^i^—C13—H13A	118.00
C4—C5—C6	127.7 (5)		
			
O1—Cu1—O2—C6	88.5 (3)	C1—N1—C5—C6	−178.0 (4)
N1—Cu1—O2—C6	−2.7 (3)	Cu1—N2—C8—C9	−178.3 (4)
N2—Cu1—O2—C6	−175.9 (3)	C12—N2—C8—C9	−0.1 (7)
O1—Cu1—O4—C7	−85.2 (3)	Cu1—N2—C12—C11	178.5 (4)
N1—Cu1—O4—C7	4.7 (3)	C8—N2—C12—C11	0.3 (7)
N2—Cu1—O4—C7	178.1 (3)	N1—C1—C2—C3	−0.4 (7)
O1—Cu1—N1—C1	93.5 (3)	C7—C1—C2—C3	−178.0 (5)
O1—Cu1—N1—C5	−86.4 (3)	N1—C1—C7—O4	6.8 (6)
O2—Cu1—N1—C1	−179.9 (3)	N1—C1—C7—O5	−171.0 (4)
O2—Cu1—N1—C5	0.1 (3)	C2—C1—C7—O4	−175.4 (5)
O4—Cu1—N1—C1	−0.6 (3)	C2—C1—C7—O5	6.8 (8)
O4—Cu1—N1—C5	179.5 (4)	C1—C2—C3—C4	1.2 (8)
O1—Cu1—N2—C8	84.0 (4)	C2—C3—C4—C5	−0.7 (8)
O1—Cu1—N2—C12	−94.2 (4)	C3—C4—C5—N1	−0.7 (7)
O2—Cu1—N2—C8	−3.9 (4)	C3—C4—C5—C6	178.7 (5)
O2—Cu1—N2—C12	178.0 (4)	N1—C5—C6—O2	−4.2 (6)
O4—Cu1—N2—C8	179.0 (4)	N1—C5—C6—O3	175.4 (4)
O4—Cu1—N2—C12	0.9 (4)	C4—C5—C6—O2	176.4 (5)
Cu1—O2—C6—O3	−175.2 (4)	C4—C5—C6—O3	−4.0 (8)
Cu1—O2—C6—C5	4.3 (5)	N2—C8—C9—C10	−0.2 (8)
Cu1—O4—C7—O5	170.2 (4)	C8—C9—C10—C11	0.2 (7)
Cu1—O4—C7—C1	−7.4 (5)	C8—C9—C10—C13	179.9 (5)
Cu1—N1—C1—C2	179.1 (3)	C9—C10—C11—C12	0.0 (7)
Cu1—N1—C1—C7	−2.9 (5)	C13—C10—C11—C12	−179.7 (5)
C5—N1—C1—C2	−0.9 (7)	C9—C10—C13—C13^i^	0.0 (8)
C5—N1—C1—C7	177.1 (4)	C11—C10—C13—C13^i^	179.7 (5)
Cu1—N1—C5—C4	−178.6 (4)	C10—C11—C12—N2	−0.2 (8)
Cu1—N1—C5—C6	2.0 (5)	C10—C13—C13^i^—C10^i^	180.0 (4)
C1—N1—C5—C4	1.5 (7)		

Symmetry codes: (i) −*x*+1, −*y*, −*z*+1.

### Hydrogen-bond geometry (Å, °)

**Table:**

*D*—H···*A*	*D*—H	H···*A*	*D*···*A*	*D*—H···*A*
O1—H1A···O6	0.82	2.10	2.669 (8)	126
O1—H1B···O2^ii^	0.82	1.99	2.809 (5)	175
O6—H6A···O3^ii^	0.82	2.31	2.919 (9)	132
O6—H6B···O3^iii^	0.82	2.06	2.851 (8)	163
C2—H2A···O1^iv^	0.93	2.54	3.348 (6)	146
C4—H4A···O3^v^	0.93	2.52	3.411 (6)	160
C8—H8A···O1^vi^	0.93	2.49	3.381 (6)	161
C9—H9A···O5^vii^	0.93	2.47	3.382 (6)	167
C13—H13A···O5^viii^	0.93	2.35	3.265 (6)	166

Symmetry codes: (ii) *x*+1, *y*, *z*; (iii) −*x*+1, −*y*+1, −*z*+2; (iv) *x*, *y*+1, *z*; (v) −*x*, −*y*+2, −*z*+2; (vi) *x*−1, *y*, *z*; (vii) *x*−1, *y*−1, *z*; (viii) −*x*+2, −*y*+1, −*z*+1.
